# Bell County Medical Association

**Published:** 1886-08

**Authors:** R. P. Talley

**Affiliations:** Sec.


### SOCIETY NOTES.

MINUTES OF BELL COUNTY MEDICAL SOCIETY, AUGUST '86.

Meeting called to order at 2 P. M. in Dr. Batte's office by Dr. Frank Allen, President. Present Drs. Allen, Armstrong, Batte, Enoch, Flint, Ghent, Hudson, Hunter, Russell, Ross and Talley.

Board of Censors reported favorably on the petition of Dr. Albert Armstrong for membership, and he was elected to membership by acclamation.

Moved and seconded that the President appoint a committee of three to take notice of the death of Dr. Burt of Austin, Texas. The President appointed the following, Doctors Batte, Ross and Talley.

The discussion of the subject selected at a previous meeting was begun by the reading of a paper on the subject by Dr. Ross. The discussion proved to be very interesting and useful.

The discussion of diagnosis, prognosis and treatment of typhoid fever was made the subject for the next meeting, and Dr. Haley was elected to open the subject with Dr. Hudson as alternate. The meeting then adjourned to meet again the first Tuesday in September.

R. P. TALLEY, Sec.

